# An artificial intelligence-derived metabolic network predicts psychosis in Alzheimer’s disease

**DOI:** 10.1093/braincomms/fcaf159

**Published:** 2025-04-25

**Authors:** Nha Nguyen, Jesus J Gomar, Jack N Truong, Janos Barbero, Patrick P Do, Andrea Rommal, Alice Oh, David Eidelberg, Jeremy Koppel, An Vo

**Affiliations:** Center for Neurosciences, The Feinstein Institutes for Medical Research, Manhasset, NY 11030, USA; The Litwin-Zucker Research Center for the Study of Alzheimer's Disease, The Feinstein Institutes for Medical Research, Manhasset, NY 11030, USA; Department of Computer Science, Erik Jonsson School of Engineering and Computer Science at The University of Texas at Dallas, Dallas, TX 75080, USA; Department of Molecular Medicine, Donald and Barbara Zucker School of Medicine at Hofstra/Northwell, Hempstead, NY 11549, USA; Manning College of Information and Computer Sciences, University of Massachusetts Amherst, Amherst, MA 01003, USA; Department of Molecular Medicine, Donald and Barbara Zucker School of Medicine at Hofstra/Northwell, Hempstead, NY 11549, USA; Center for Neurosciences, The Feinstein Institutes for Medical Research, Manhasset, NY 11030, USA; Center for Neurosciences, The Feinstein Institutes for Medical Research, Manhasset, NY 11030, USA; Department of Molecular Medicine, Donald and Barbara Zucker School of Medicine at Hofstra/Northwell, Hempstead, NY 11549, USA; The Litwin-Zucker Research Center for the Study of Alzheimer's Disease, The Feinstein Institutes for Medical Research, Manhasset, NY 11030, USA; Department of Molecular Medicine, Donald and Barbara Zucker School of Medicine at Hofstra/Northwell, Hempstead, NY 11549, USA; Center for Neurosciences, The Feinstein Institutes for Medical Research, Manhasset, NY 11030, USA; Department of Molecular Medicine, Donald and Barbara Zucker School of Medicine at Hofstra/Northwell, Hempstead, NY 11549, USA

**Keywords:** Alzheimer’s disease psychosis, biomarker, convolutional neural network, explainable AI, metabolic brain networks

## Abstract

The delusions and hallucinations that characterize Alzheimer’s disease psychosis (AD + P) are associated with violence towards caregivers and an accelerated cognitive and functional decline whose management relies on the utilization of medications developed for young people with schizophrenia. The development of novel therapies requires biomarkers that distinguish AD + P from non-psychotic Alzheimer’s disease. We investigated whether there might exist a brain metabolic network that distinguishes AD + P from non-psychotic Alzheimer’s disease that could be used as a biomarker to predict and track the course of AD + P for use in clinical trials. Utilizing F-18 fluorodeoxyglucose positron emission tomography scans from cohorts of cognitively healthy elderly (*N* = 174), those with Alzheimer’s disease without psychosis (*N* = 174) and those with AD + P (*N* = 88) participating in the Alzheimer’s Disease Neuroimaging Initiative study, we employed a convolutional neural network to identify and validate the Alzheimer’s Psychosis Network. We analysed network progression, clinical correlations and psychosis prediction using expression scores and network organization using graph theory. The Alzheimer’s Psychosis Network accurately distinguishes AD + P from controls (97%), with increasing scores correlating with cognitive decline. The Alzheimer’s Psychosis Network–based approach predicts psychosis in Alzheimer’s disease with 77% accuracy and identifies specific brain regions and connections associated with psychosis. Alzheimer’s Psychosis Network expression was found to be associated with increased cognitive and functional decline that characterizes AD + P. The increased metabolic connectivity between motor and language/social cognition regions in AD + P may drive delusions and agitated behaviour. Alzheimer’s Psychosis Network holds promise as a biomarker for AD + P, aiding in treatment development and patient stratification.

## Teaser

Nguyen *et al*. report that an artificial intelligence–derived metabolic brain network identifies psychosis in Alzheimer’s disease with a high degree of accuracy. This Alzheimer’s disease psychosis network’s expression also predicts longitudinal cognitive and functional outcomes, suggesting potential as a biomarker in novel therapy development for psychotic Alzheimer’s disease.

## Graphical Abstract

**Graphical Abstract:**
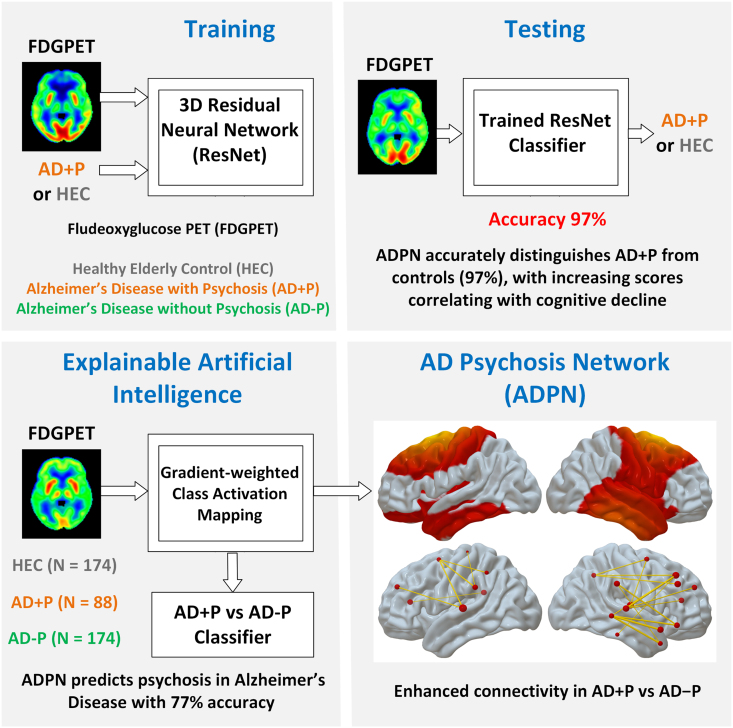

## Introduction

There is evidence that the emergence of psychosis in Alzheimer’s disease (AD + P), manifested by delusional beliefs and/or hallucinatory experiences, is the syndromal expression of a distinct pathophysiologic subtype with a unique clinical course that can be distinguished from non-psychotic Alzheimer’s disease.^1,2^ AD + P is associated with aggressive behaviour towards caregivers and leads to higher rates of placement outside of the home in skilled nursing facilities.^1^ Longitudinal studies have consistently found that those with Alzheimer’s disease who will develop psychosis over the course of disease (when compared with those with Alzheimer’s disease who never experience psychosis) have a more precipitous trajectory of decline that often precedes the onset of the delusions and hallucinations and is associated with a hastened mortality.^3-9^ As this suggest a more aggressive form of neurodegenerative disease, neuropathological correlates of this rapid decline observed in psychotic Alzheimer’s disease have been investigated by our group and others, and results from histologic and biochemical characterization of brain tissue^10-12^; cerebrospinal fluid analysis^13^ and tau PET^14^ and very recently plasma^15^ have all implicated the burden of tau pathology as a mediator of decline. Of the relevant biomarkers of Alzheimer’s disease that have been organized into an explanatory framework that comprises amyloid deposition/tau pathology/neurodegeneration (‘A/T/N’), hypometabolism as an indication of neurodegeneration has the strongest association with the scope of cognitive impairment.^16^ When compared with those who do not experience psychosis in Alzheimer’s disease, increased regional impairments in cortical metabolic activity that likely reflect focal neurodegeneration have been reported in those with delusions and hallucinations over the course of Alzheimer’s disease, although no studies have directly compared the strength of this association with the contribution of other Alzheimer’s disease biomarkers.^17,18^

Network analysis, a technique employed to map covariance topographies in functional neuroimaging that represent patterns of connectivity,^19^ has been used to identify the abnormal neural circuitry that is a consequence of neurodegeneration, and that is associated with the expression of unique cognitive and motor phenotypes.^20^ These approaches have clinical value in aiding differential diagnosis via the determination of the consistency of established disease-network patterns with individual scans and in predicting response to treatment for conditions such as Parkinson’s disease.^20^ Analytic approaches employing metabolic maps of glucose utilization with fluorodeoxyglucose positron emission tomography (FDG PET) and blood oxygenation-dependent signals have been used successfully to identify and characterize patterns in Alzheimer’s disease, Parkinson’s disease and most recently Dementia with Lewy Bodies.^20^ The Alzheimer’s disease–related pattern of network dysfunction has been mapped with FDG PET and comprises metabolic reductions in precuneus, posterior cingulate and canonical temporoparietal regions of Alzheimer’s disease significance that co-vary with metabolic increases in the pons and sensorimotor cortex—a region that is generally spared of Alzheimer’s disease neuropathology.^21-23^ The quantification of Alzheimer’s disease–related pattern expression has been shown to diagnostically differentiate Alzheimer’s disease from other neurodegenerative conditions and correlates with the degree of cognitive impairment, making it a useful tool for neurodegenerative disease work-up and a potentially valuable biomarker of disease progression that could be used to track response to emerging Alzheimer’s disease treatments.^22,23^

Treatment of AD + P currently relies on antipsychotic medications developed for the treatment of schizophrenia that are modestly effective in AD + P,^24-27^ especially in those with more severe symptoms^28^; however, their use in dementia is associated with an increased risk of death.^27^ The ability to predict the likelihood of antipsychotic treatment response in AD + P could ameliorate the troublesome risk–benefit ratio by helping clinicians limit exposure to those most likely to respond; of equal importance, any predictive biomarkers could function as surrogate markers of treatment response in AD + P clinical trials aiding the development of novel therapies. The identification of a neural network could be a first step in the process of predictive AD + P biomarker development. In support of this approach, neural networks have been identified in schizophrenia that predict antipsychotic treatment response.^29-32^ As an early increased burden of cognitive impairment predicts the onset of psychosis in Alzheimer’s disease,^5,7^ and as network analyses utilizing metabolic patterns from FDG PET correlated with cognitive dysfunction have been successful in building maps that distinguish neurodegenerative diseases, we sought to develop a novel AD + P biomarker by investigating whether an Alzheimer’s disease psychosis network might exist that distinguishes those who become psychotic over the course of Alzheimer’s disease from those who do not.

While much is known about the regional patterns of metabolic impairment that characterize the cognitive and motor deficits of neurodegenerative disease enabling the targeted use of tools for dimensionality reduction in neural network design, the topography of focal impairments in the common psychiatric manifestations of these illnesses are more elusive. However, recent advances in artificial intelligence (AI)^33,34^ have revolutionized the study of disease-related networks in neuroimaging, particularly revealing patterns that elude traditional methods. AI has been used for diagnostic and prognostic neuroimaging in dementia.^35^ In Alzheimer’s disease, deep learning has demonstrated utility in providing a comprehensive clinical diagnostic assessment that integrates neuroimaging data with symptomatology and neurocognitive assessments.^36,37^ In this context, deep learning could be employed on neuroimaging data to identify and validate specialized brain networks associated with psychiatric symptoms, offering predictions for diagnostic categories or specific clinical features in patients. To this aim, in the present study, we apply convolutional neural networks (CNNs) within an explainable AI framework to FDG PET in an Alzheimer’s disease psychosis cohort drawn from the Alzheimer’s Disease Neuroimaging Initiative (ADNI) database. We identify and characterize a novel metabolic map of AD + P, the Alzheimer’s Disease Psychosis Network (ADPN) and employing graph theory, we delve into the ADPN organization. Additionally, we assess the network progression through longitudinal scans and examine the correlations between clinical measures and expression scores. Finally, the ADPN is employed to predict psychosis in Alzheimer’s disease, and we explore the specific brain regions and connections that differentiate the Alzheimer’s disease groups with/without psychosis.

## Materials and methods

### Study design

**Figure 1:**
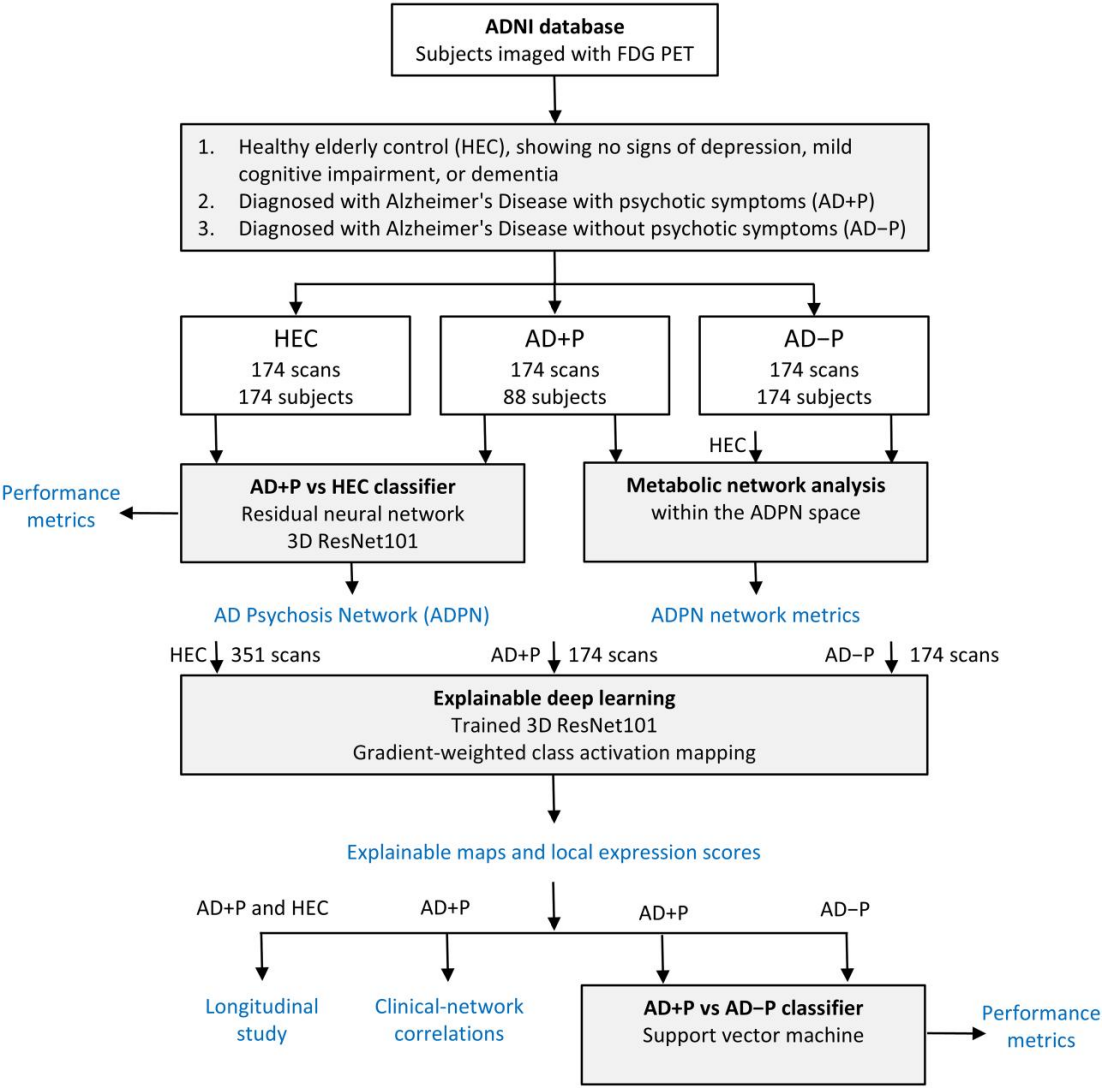
**Flowchart of the study design.** We developed a 3D residual neural network to characterize the Alzheimer’s disease psychosis network using FDG PET scans obtained from the ADNI database. After the training phase, we employed an explainable deep learning technique to generate explainable maps and expression scores for each scan. The AD + P expression scores were then utilized to assess the rate of network progression through longitudinal scans, evaluate correlations with clinical measures and predict psychosis in AD. Additionally, metabolic network analysis was conducted within the ADPN space to identify differences in network organization among the groups. ADNI, Alzheimer’s Disease Neuroimaging Initiative.

The study design flowchart is presented in Fig. 1. Our analysis involved 699 FDG PET scans from a normal control group and two Alzheimer’s disease groups, one with psychosis and one without, obtained from the ADNI database. Initially, we employed a residual neural network^38^ to identify and validate the ADPN. Subsequently, we utilized an explainable deep learning technique^39^ to generate explainable maps and compute network expression scores. These scores were then used to assess the rate of network progression through longitudinal scans and examine correlations with clinical measures including clinical dementia rating scale sum of boxes (CDRSB) and mini-mental state examination (MMSE). In the next analysis, we constructed an ADPN-based classifier for predicting psychosis in Alzheimer’s disease, incorporating expression scores and support vector machine. The performance metrics of these classifiers were compared with a conventional PET-based approach. Additionally, we conducted graph theoretical analysis within the ADPN space to reveal differences in network organization across the three groups.

### Participants

The participants’ data and their FDG PET scans were retrieved from the ADNI database (https://adni.loni.usc.edu) on November 21, 2022. To assess the presence or absence of psychotic symptoms, the first two items (delusions and hallucinations, NPIA, NPIB) of the 12-item Neuropsychiatric Inventory were utilized, in accordance with consensus criteria for psychosis in dementia.^2^

ADNI Alzheimer’s disease participants were categorized as Alzheimer’s disease with psychosis (AD + P) if their NPIA or NPIB score over the study period was greater than zero and as Alzheimer’s disease without psychosis (AD−P) if their NPIA and NPIB scores were zero. Within the ADNI database, 88 Alzheimer’s disease participants who developed psychosis during the study were identified, with 174 valid FDG PET scans at any timepoint (baseline, 6, 12 and 24 months). Additionally, 174 age- and gender-matched Alzheimer’s disease participants who did not develop psychosis and 174 age- and gender-matched healthy elderly controls (HEC) who showed no signs of depression, mild cognitive impairment or dementia were selected, with their baseline scans included. Participants from ADNI cohort underwent imaging at different sites, as detailed on the ADNI website (https://adni.loni.usc.edu/data-samples/adni-data/neuroimaging/pet/). Co-registered, averaged standardized images with uniform voxel size and resolution were obtained from ADNI.

Demographic and clinical characteristics of the study participants were provided in Table 1. For the longitudinal study, we identified 26 AD + P participants scanned at baseline, 6 months and 12 months, and 29 AD + P participants scanned at baseline and 24 months, along with 59 HEC participants scanned at these four timepoints. The ADNI protocol received approval from the regional ethical committees of all participating institutions, and written informed consent was provided by all study participants.

**Table 1:** Demographic and clinical characteristics

	HEC	AD + P	AD−P
Subjects	174	88	174
Scans	351	174	174
Age (years)	74.4 ± 5.8^b^	75.0 ± 7.5	74.5 ± 8.6
Sex F, %	75, 43%	38, 43%	70, 40%
Education (years)	16.5 ± 2.6	15.2 ± 3.1	15.4 ± 2.7
MMSE	28.9 ± 1.3	23.0 ± 2.3	23.0 ± 2.3
CDRSB	0.03 ± 0.12	4.8 ± 1.6	4.6 ± 1.6
APOE4 (Y, %)	51, 29%	63, 72%	63, 60%
NPIA = 0 & NPIB = 0^a^	351	0	174
NPIA > 0 and/or NPIB > 0 (at scan visit/other visits)	0	82/92	0

AD + P, AD with psychosis; AD, dementia due to Alzheimer’s disease; AD−P, AD without psychosis; APOE4, a specific allele of the apolipoprotein E gene; CDRSB, clinical dementia rating scale sum of boxes; F, female; HEC, healthy elderly control; M, male; MMSE, mini-mental state examination; N, no; P, psychosis; Y, yes.

^a^NPIA and NPIB are the first 2 items of the 12-item Neuropsychiatric Inventory (NPI-Q).^2^

^b^All the data are presented as mean ± SD.

### Image pre-processing

FDG PET scans were registered to a standard Montreal Neurological Institute (MNI)-based PET template with a resolution of 2 mm and a matrix size of 91 × 109 × 91 and were smoothed with an isotropic Gaussian kernel (8 mm) in all directions to improve the signal-to-noise ratio using the FMRIB library (http://www.fmrib.ox.ac.uk/fsl/). All FDG PET images were intensity normalized to the mean value of cerebellum.

### ADPN identification and validation

#### 3D residual neural network classifier

In this analysis, we implemented a 3D CNN (Supplementary Fig. 1) based on a 2D residual neural network (ResNet101)^38^ using the Deep Learning Toolbox in MATLAB R2023a. The data input into the CNN is a 3D image with a matrix size of 91 × 109 × 91, which is the size of FDG PET volume after image preprocessing. The ResNet101, initially pre-trained with a depth of 101 layers on a large dataset of over one million 2D images from the ImageNet database, extracted useful features and patterns from a diverse set of images. The learning parameters from the pre-trained model were incorporated as bias parameters in our 3D framework, except for the first layer, which was modified to support the input data size of 91 × 109 × 91. To adapt the model for 3D images, we replaced the 2D convolutional filters with 3D filters and adjusted the sizes of convolutional and max pooling layers accordingly, and the parameters of the proposed 3D ResNet101 are detailed in Supplementary Table 1 and Supplementary Fig. 1. A subject-independent random split was performed, allocating 80% of the AD + P subjects to the training set (70 subjects, *N* = 142 scans) and 20% to the testing set (18 subjects, *N* = 32 scans). Then, we matched them to an equal number of scans from the HEC group. The 3D ResNet101 was trained and fine-tuned on the FDG PET images from the AD + P and HEC training sets to identify the ADPN and validated on their respective testing sets. The performance of the ADPN classifier was then compared with that of a conventional PET-based classifier,^40^ achieved through 95 FDG PET regions of interest based on the AAL atlas,^41^ and a support vector machine.

#### Explainable deep learning

##### Gradient-weighted class activation mapping

The 3D ResNet101 model classifies each scan into one of the prediction classes (e.g. AD + P or HEC) without localizing or identifying specific regions that contribute to this classification. To identify these contributing brain regions, we employed Gradient-weighted class activation mapping (Grad-CAM),^39^ an explainable deep learning technique, using Deep Learning Toolbox in MATLAB 2023a after the training phase. This method generates a 3D explainable map for each FDG PET scan by calculating the gradients of the classification score with respect to the final convolutional layer of the trained 3D ResNet101 network, highlighting the areas most influential in the final classification decision. Areas with higher gradient values indicate where the final score is most affected by the data.

##### Connecting Grad-CAM and ROI-based analysis

To link global classification (3D CNN output) with region-specific insights, we computed the mean value of the Grad-CAM explainable map within each predefined region of interest using the AAL atlas (95 ROIs). This mean value, termed the ‘local expression score’, quantifies each region's contribution for downstream analyses, including longitudinal study, clinical-network correlation and AD + P prediction from AD−P. Integrating Grad-CAM into the downstream analysis pipeline improves interpretability by associating CNN predictions with specific brain regions, providing biologically meaningful insights while maintaining the advantages of the 3D CNN's global classification. To examine regional differences in explainable maps between AD + P and HEC, local expression scores for each of the 95 AAL regions were compared. Brain regions specific to the difference between the two groups were determined when their scores were significantly different (*P* < 0.05, Bonferroni correction; training dataset). In which, brain regions specific to each group (HEC or AD + P) are identified based on a significantly greater expression score compared with the other group (*P* < 0.05 and Bonferroni correction, training dataset). The HEC or ADPN expression score is the average of local expression scores across regions specific to that respective group. These scores were *z*-scored with reference to the HEC distribution of training set. The difference in these expression scores between the two groups was examined in both the training and testing sets at baseline. For visualization purpose, a group explainable map was computed for each group by averaging the explainable maps of subjects within that group.

#### Longitudinal analysis in AD + P space

To assess the rate of network progression in AD + P subjects, we utilized longitudinal FDG PET scans from 26 participants with AD + P, scanned at baseline, 6 months and 12 months, and 29 AD + P participants scanned at baseline and 24 months. Additionally, 59 HEC participants with normal cognitive function underwent longitudinal FDG PET scans at these four timepoints for comparative longitudinal analysis. This involved computing the AD + P expression score in individual explainable map obtained at baseline and at 6-, 12- and 24-months follow-up. Changes in the AD + P expression score from baseline were then evaluated at each follow-up.

#### Clinical-AD psychosis network correlations

We applied local expression scores from regions specific to AD + P groups to a linear regression model as described in study by Vo *et al*.^42^ and Schindlbeck *et al.*^43^ using the Machine Learning Toolbox in MATLAB R2023a to predict CDRSB for AD + P subjects. Half of the data was utilized for training, and the remaining half was used for testing. Subsequently, we assessed the correlations between the prediction score and both CDRSB and MMSE.

### ADPN predicts psychosis in Alzheimer’s disease

To explore the difference between AD + P and AD−P in the ADPN network, explainable maps for AD−P scans were generated using Grad-CAM and the trained ResNet101. Subsequently, we performed prospective computation of local expression scores for AD−P patients.

We developed an ADPN-based classifier to predict AD + P (*n* = 174 scans) and AD−P (*n* = 174 scans) using local expression scores from the AD + P key regions and a support vector machine. Validation was conducted using 5-fold cross-validation. The performance of the ADPN-based classifier was compared with that of a conventional PET-based classifier, achieved through FDG PET features of the same regions, and a support vector machine.

To examine regional differences in explainable maps between AD + P and AD−P, local expression scores for each of the 95 AAL regions were compared. Key regions specific to the difference in explainable maps between the two groups were determined when their expression scores were significantly different (*P* < 0.05, Bonferroni correction; baseline).

### ADPN network organization

In this analysis, we applied graph-theory method^44-48^ to investigate differences in ADPN network organization, including network connectivity and metrics across three groups. The ADPN was parcellated into 95 regions of interest (nodes) using the AAL atlas, as described previously.^41,49^ Key regions specific to the ADPN were then selected for further network analysis.

For each node, we computed normalized metabolic activity based on FDG PET scans. The metabolic data from each group were utilized to construct node-to-node correlation matrices separately for HEC, AD + P and AD−P. We generated 100 bootstrap samples for each group, calculating pairwise nodal correlation coefficients (Pearson correlations) for each iteration. The median values of the 100 bootstrap correlation estimates were used to create an adjacency matrix for the network in each group. These calculations were performed using the Machine Learning Toolbox in MATLAB R2023a.

#### Network connectivity

We examined changes in metabolic connectivity (enhanced/reduced or gained/lost) between two groups by comparing all the connection pairs using a method described elsewhere.^45^ A connection was considered enhanced or reduced relative to a reference if either the connection or the reference exceeded |*r*| ≥ 0.6 (*P* < 0.05) and |*Δr*| > 0.2 (*P* < 0.05, permutation test, 1000 iterations). Validation of connections meeting these criteria was performed using 100 bootstrap samples (*P* < 0.05, Bonferroni correction).

#### Network metrics

To assess group differences in network organization within the ADPN space, we computed the following metrics using the Brain Connectivity Toolbox^50^ and an in-house script (MATLAB R2023a):

1. Mean degree centrality: Measures the average number of connections that each node in the network has.
2. Clustering coefficient: Indicates the number of triangles in a graph or how neighbours of a node are connected to each other.
3. Characteristic path length: Represents the shortest path length between two nodes averaged over all pairs of nodes. A high characteristic path length implies less efficient information transfer through the network.
4. Small-worldness: This is the ratio of clustering coefficient to characteristic path length, normalized to corresponding parameters from an equivalent random graph. It quantifies the ratio of segregation to integration of information sources in the network space.

We present network metrics over a range of connectivity thresholds ranged from *r* = 0.3 to 0.6, at 0.05 increments as described previously. This presentation aims to illustrate the robustness of group differences in a given metric, extending beyond two or three adjacent levels. In terms of network visualization, graphs were presented at the threshold (Level 7, *r* = 0.6) using Surf Ice (version 10/6/2021; https://www.nitrc.org/projects/surfice/).

### Statistical analysis

We compared expression scores, metabolic activity, demographic and clinical characteristics between groups using Student’s *t*-test. Chi-square test was used to assess the ApoE4 genotype between the AD−P and AD + P groups. Group differences and changes in expression scores during the follow-up period (6, 12 and 24 months) were assessed using a general linear model, with *post hoc* Bonferroni tests for pairwise comparisons of time points relative to baseline. Pearson’s correlations were used to evaluate the associations between ADPN prediction scores and clinical measures (CDRSB and MMSE). In the graph analysis, the bootstrapped data were used to assess group differences in network metrics. A general linear model across graph thresholds, followed by *post hoc* Bonferroni tests, was utilized to evaluate group differences in each graph metrics. These analyses were performed using MATLAB R2023a. Results were considered significant for *P* < 0.05, with Bonferroni correction applied for multiple comparisons.

## Results

### Demographics

AD + P (*N* = 88) and AD−P (*N* = 174) groups were balanced for age (75±7.5 versus 74.5±8.6 years), education (15.2±3.1 versus 15.4±2.7 years), sex (43% versus 40% female) and baseline MMSE (23±2.3 versus 23±2.3). As in previous reports,^51^ AD + P subjects were more likely to carry the ApoE4 genotype (72%) than AD−P subjects (60%), *P* = 0.16 (Chi-square test).

### Alzheimer’s disease psychosis network

**Figure 2:**
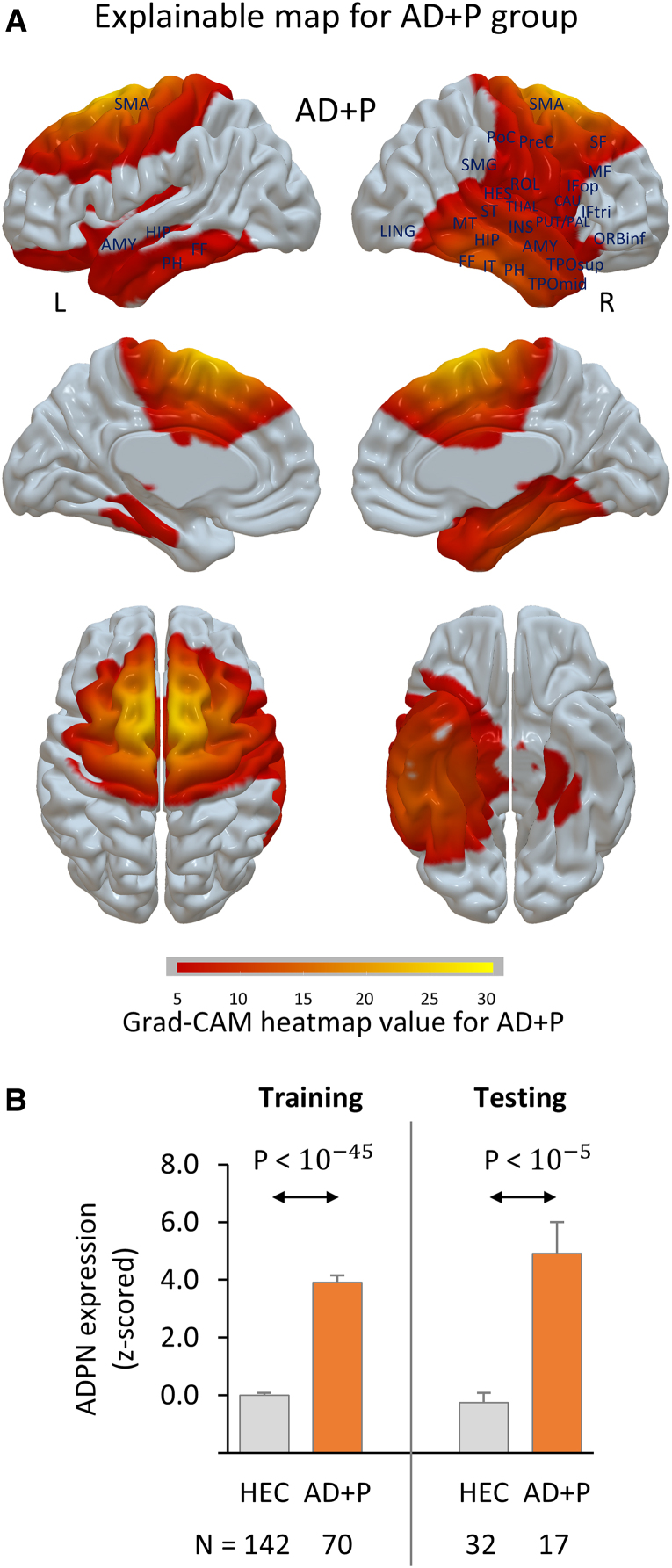
**Alzheimer’s disease psychosis network (ADPN).** The ADPN was identified from 142 AD + P and 142 HEC FDG PET scans and subsequently validated on a dataset consisting of 32 AD + P and 32 HEC scans. (**A**) Explainable map for the AD + P group (*N* = 87), computed by averaging the explainable maps of AD + P subjects. (**B**) The ADPN expression score at baseline exhibited an elevation in AD + P compared with the HEC subjects [training set: T(210) = 19.2, *P* < 10^−45^; testing set: T(47) = 5.6, *P* < 10^−5^). Student’s *t*-test was used to examine the difference in expression scores between the two groups. AD + P, AD with psychosis; AMY, amygdala; CAU, caudate; FF, fusiform; Grad-CAM, gradient-weighted class activation; HEC, healthy elderly controls; HES, Heschl; HIP, hippocampus; IFop, inferior frontal operculum; IFtri, inferior frontal triangularis; INS, insula; IT, inferior temporal; L, left; LING, lingual; MF, middle frontal; MT, middle temporal; ORBinf, inferior frontal orbital; PAL, pallidum; PH, parahippocampal; PoC, postcentral; PreC, precentral; PUT, putamen; R, right; ROL, rolandic operculum; SF, superior frontal; SMA, supplementary motor area; SMG, supramarginal; ST, superior temporal; THAL, thalamus; TPOmid, middle temporal pole; TPOsup, superior temporal pole.

The ADPN classifier was trained using FDG PET data from 142 AD + P and 142 HEC scans and subsequently tested on a dataset consisting of 32 AD + P and 32 HEC scans. It exhibited a higher accuracy (96.9%) compared with the conventional PET-based approach (92.2%), achieved through features from FDG PET regions of interest, and a support vector machine.^40^ Additional performance metrics of the ADPN, including sensitivity, specificity, precision and F1 score, also outperformed those of the conventional classifier (Supplementary Table 2). The ADPN network so derived identified key regions that exhibit significantly different expression scores in AD + P and HEC groups. These regions included (i) the prefrontal cortex; (ii) the precuneus, angular and supramarginal gyrus, superior and inferior parietal cortex; (iii) the temporal pole, middle temporal cortex and the primary auditory cortex inclusive of Heschl’s gyri and the superior temporal cortex; (iv) the visual cortex including lingual gyrus, middle and superior occipital cortex; (v) limbic areas such as the hippocampus, parahippocampal gyrus, amygdala, thalamus and cingulate gyrus and (vi) the striatum and sensorimotor cortex (Table 2, Supplementary Table 3). Specifically, the explainable map for AD + P that comprises the ADPN includes all brain regions with significantly elevated expression scores compared with the HEC (graphic representation Fig. 2A). The average ADPN expression score calculated as a composite of these regions was significantly higher in AD + P compared with the HEC subjects (Fig. 2B, training set: *P* < 10^−45^; testing set: *P* < 10^−5^). Alternatively, the explainable map for the HEC group (Supplementary Fig. 2) revealed specific brain regions (Supplementary Table 3) with significantly higher expression scores than those of AD + P. The average expression scores across these regions were significantly higher in HEC compared with the AD + P subjects (Supplementary Fig. 2, bottom; training set: *P* < 10^−60^; testing set: *P* < 10^−8^).

**Table 2:** The AD psychosis network exhibits the significant difference in expression scores between AD + P and HEC

Brain regions specific to AD + P (Expression score in AD + P > HEC)		
Brain region	Abbreviation	AAL ID
Precentral_R	PreC	2
Frontal_Sup_R	SF	4
Frontal_Mid_R	MF	8
Frontal_Inf_Oper_R	IFop	12
Frontal_Inf_Tri_R	IFtri	14
Frontal_Inf_Orb_R	ORBinf	16
Rolandic_Oper_R	ROL	18
Supp_Motor_Area_LR	SMA	19–20
Insula_R	INS	30
Hippocampus_LR	HIP	37–38
ParaHippocampal_LR	PH	39–40
Amygdala_LR	AMY	41–42
Lingual_R	LING	48
Fusiform_LR	FF	55–56
Postcentral_R	PoC	58
SupraMarginal_R	SMG	64
Caudate_R	CAU	72
Putamen_R	PUT	74
Pallidum_R	PAL	76
Thalamus_R	THAL	78
Heschl_R	HES	80
Temporal_Sup_R	ST	82
Temporal_Pole_Sup_R	TPOsup	84
Temporal_Pole_Mid_R	TPOmid	88
Temporal_Mid_R	MT	86
Temporal_Inf_R	IT	90
Cerebellum_LR	CRBL	91–92
Vermis	Ver	93
Pons_LR	Pons	94–95

AD + P, AD with psychosis; AAL, automated anatomical labeling brain atlas^41^; HEC, normal control; L, left; R, right.

#### Longitudinal study of AD + P

**Figure 3:**
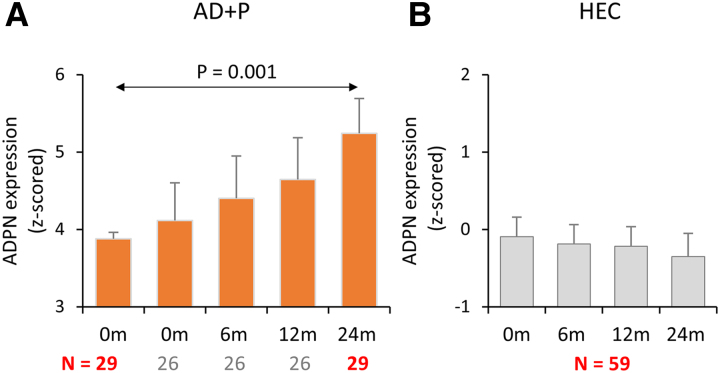
**Longitudinal changes in the ADPN expression scores**. (**A**) In AD + P patients, expression scores increased significantly over time [*F*(3, 48) = 5.8, *P* = 0.002, *N* = 17]. *Post hoc* Bonferroni tests revealed a significant increase at 24 months compared with baseline (*P* = 0.001, *N* = 29), but not at 6 or 12 months. (**B**) In contrast, ADPN expression scores in healthy controls did not change significantly over time [*F*(3, 162) = 0.69, *P* = 0.56, *N* = 55]. Group differences and changes in expression scores during the follow-up period (6, 12 and 24 months) were assessed using a general linear model, with *post hoc* Bonferroni tests for pairwise comparisons of time points relative to baseline. AD + P, AD with psychosis; ADPN, Alzheimer’s disease psychosis network; HEC, healthy elderly controls.

As neurodegeneration is a dynamic process which would be expected to impact the psychosis-specific network score in individuals over time, and as disease-specific network values have previously been shown to increase over time in Alzheimer’s disease and Parkinson’s disease in tandem with clinical decline,^19,20^ ADPN scores were assessed longitudinally in an AD + P compared with a HEC group in which scans were available to 24 months. As expected, the ADPN expression scores were significantly higher at baseline in AD + P subjects (*P* < 10^−9^, Bonferroni corrected) compared with HEC subjects. Over the follow-up period, comparisons between groups revealed that ADPN expression scores were significantly increased in the AD + P group (*N* = 29) with larger deviations in values from the HEC group (*N* = 59) at each successive time point (Fig. 3; 6 months: *P* < 10^−11^; 12 months: *P* < 10^−12^; 24 months: *P* < 10^−14^; Bonferroni corrected). Within the AD + P group, expression scores increased significantly at 24 months compared with baseline (*P* = 0.001, *N* = 29, Bonferroni corrected), but not at 6 or 12 months (Fig. 3). As expected, ADPN scores did not increase in the HEC group over time.

#### Clinical-AD psychosis network correlations

**Figure 4:**
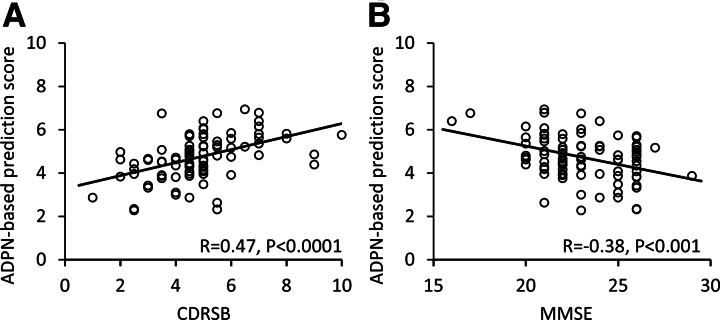
**Clinical-AD psychosis network correlations**. In AD + P patients, the ADPN-based prediction scores significantly correlated with (**A**) Clinical dementia rating scale sum of boxes (CDRSB) (*N* = 87, training and testing), and (**B**) Mini-mental state examination (MMSE) (*N* = 87, testing). Pearson’s correlations were used to evaluate the associations between ADPN prediction scores and clinical measures (CDRSB and MMSE). Each data point represents the true and predicted scores for each subject scanned at baseline. AD + P, AD with psychosis; ADPN, Alzheimer’s disease psychosis network.

Psychosis in Alzheimer’s disease has been associated with more robust impairment and a rapid cognitive and functional decline.^14,18,52^ For this reason, we sought to determine whether ADPN network scores would correlate with impairments reflected in CDR and MMSE ratings. Significant correlations with increasing CDRSB scores reflecting greater functional impairment were observed for ADPN-based prediction scores computed in the training set (*R* = 0.49, *P* < 0.001, *N* = 43), testing set (*R* = 0.47, *P* < 0.005, *N* = 44) as well as the entire AD + P dataset (*R* = 0.47, *P* < 0.0001, *N* = 87, Fig. 4A). The correlation between the prediction score and MMSE scores were also examined in this cohort, revealing a significant network relationship with declining MMSE (*R* = −0.38, *P* < 0.001, *N* = 87, Fig. 4B) reflecting deteriorating cognition.

### ADPN predicts psychosis in Alzheimer’s disease

**Figure 5:**
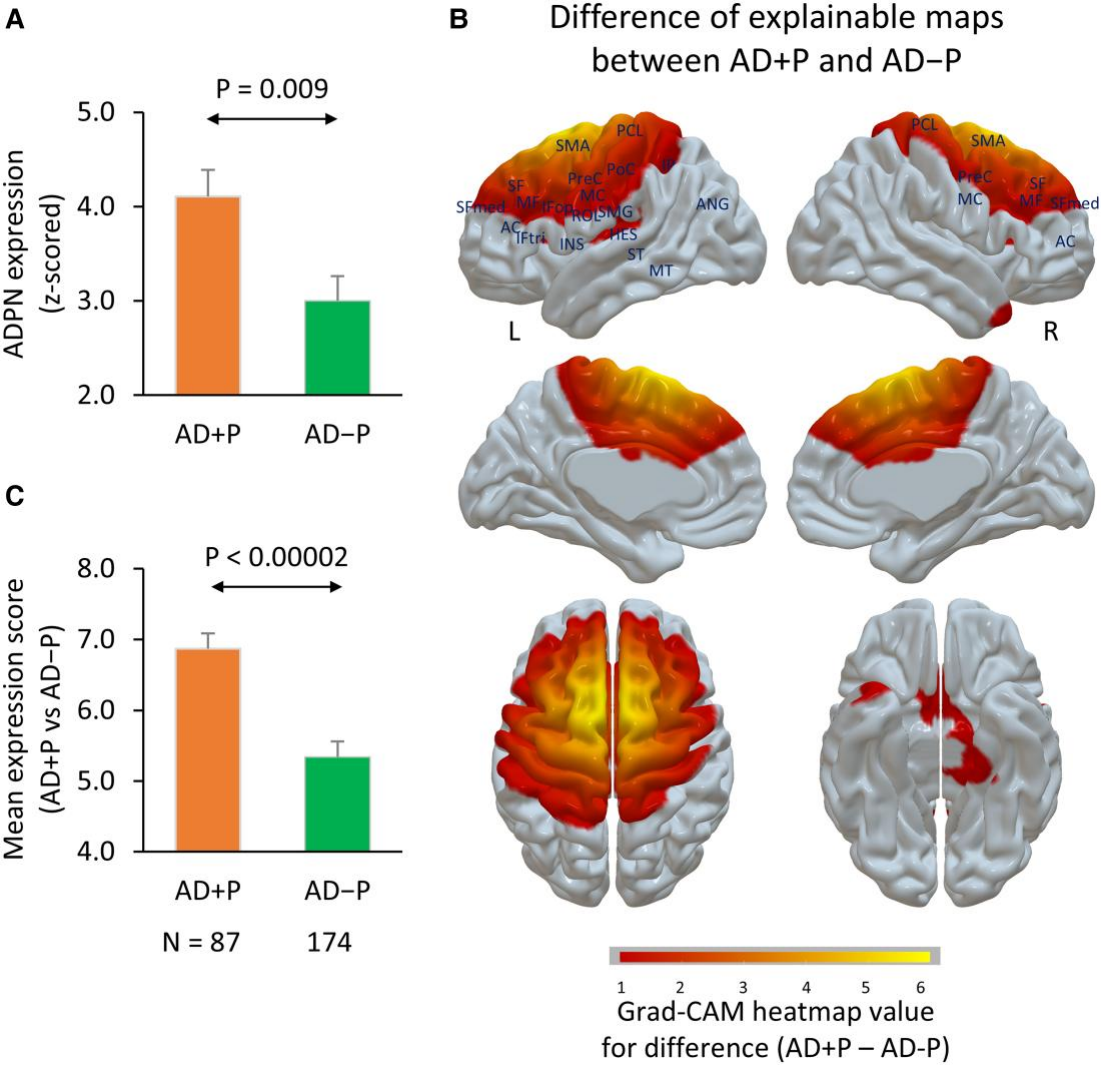
**Differences between AD + P and AD−P in the ADPN at baseline**. (**A**) The AD + P group (*N* = 87) showed a significantly higher ADPN score compared with AD−P (*N* = 174) (T(259) = 2.6, *P* = 0.009). (**B**) Difference in group explainable maps between AD + P (*N* = 87) and AD−P (*N* = 174). When compared with the AD−P patients, the AD + P exhibited brain regions with significantly higher expression scores (*P* < 0.05, Student’s *t*-test, Bonferroni correction; see Supplementary Table 5), including (i) the prefrontal cortex, (ii) the inferior parietal cortex, angular and supramarginal gyrus, (iii) the primary auditory cortex inclusive of Hesch’s gyri and the superior temporal cortex, (iv) the anterior and middle cingulate gyrus, (v) the insula and (vi) the supplemental motor area, the primary motor (M1) and somatosensory (S1) cortex. (**C**) The average expression score across these regions exhibited a significant elevation [T(259) = 4.4, *P* < 0.00002) in AD + P (*N* = 87)] compared with the AD−P (*N* = 174). Student’s *t*-test was used to examine the difference in expression scores between the two groups in **A** and **C**. AC, anterior cingulum; AD + P, AD with psychosis; AD−P, AD without psychosis; ADPN, Alzheimer’s disease psychosis network; ANG, angular; Grad-CAM, gradient-weighted class activation; HES, Heschl; IFop, inferior frontal operculum; INS, insula; IP, inferior parietal; L, left; MC, middle cingulum; MF, middle frontal; MT, middle temporal; PCL, paracentral lobule; PoC, postcentral; PreC, precentral; R, right; ROL, rolandic operculum; SF, superior frontal; SFmed, medial superior frontal; SMA, supplementary motor area; SMG, supramarginal; ST, superior temporal.

Network scores were utilized in a classification paradigm to determine whether the ADPN could discriminate AD + P from AD−P. As AD + P and AD−P share neurogenerative commonality, it was expected that the divergence would be considerably less than what was observed in comparison with the HEC group. The novel ADPN-based classifier, incorporating network score features, achieved a superior accuracy (77%) compared with the conventional PET-based classifier utilizing features from FDG PET regions of interest (68.4%; Supplementary Table 4) in distinguishing between AD + P and AD−P patients. Additional performance metrics, including sensitivity, specificity, precision and F1 score for the datasets of AD + P and AD−P, are detailed in Supplementary Table 4. The ADPN exhibited a greater sensitivity (86.2%) in detecting the significant difference between AD + P and AD−P compared with the conventional approach (67.8%). At baseline, the AD + P group showed a significantly higher ADPN score compared with AD−P (*P* = 0.009, Fig. 5A).

When compared with AD−P subjects, AD + P subjects were distinguished by baseline elevations in regionally-specific expression within the ADPN (*P* < 0.05, Bonferroni correction; Supplementary Table 5). These regions included (i) the prefrontal cortex, (ii) the inferior parietal cortex, angular and supramarginal gyrus, (iii) the primary auditory cortex such as Heschl’s and the superior temporal cortex, (iv) the anterior and middle cingulate gyrus, (v) the insula and (vi) the supplemental motor area (SMA), the primary motor (M1) and somatosensory (S1) cortex. The average expression score across these regions within the ADPN was significantly higher in the AD + P group when compared with AD−P, suggesting that these are the non-overlapping psychosis-relevant regions (*P* < 2.10^−5^, Fig. 5B and C), suggesting that ADPN quantification may be a useful biomarker for psychosis in Alzheimer’s disease.

### Alternations in the ADPN network organization: connectivity

We sought to identify any functional connections altered in the AD + P group within the ADPN network space in comparison to HEC and AD−P groups. When compared with HEC, AD + P exhibited a notable loss of metabolic connections as illustrated in Supplementary Fig. 3 and Supplementary Table 6. In AD + P, connections linking the anterior cingulate cortex (ACC) and amygdala to the angular gyrus was reduced in AD + P relative to HEC. Other connections linking (i) the prefrontal cortex; (ii) the auditory cortex such as Heschl’s and the superior temporal cortex; (iii) limbic areas such as the hippocampus and parahippocampal gyrus and (iv) the primary motor cortex to the visual cortex were all decreased relative HEC (Supplementary Table 6).

**Figure 6:**
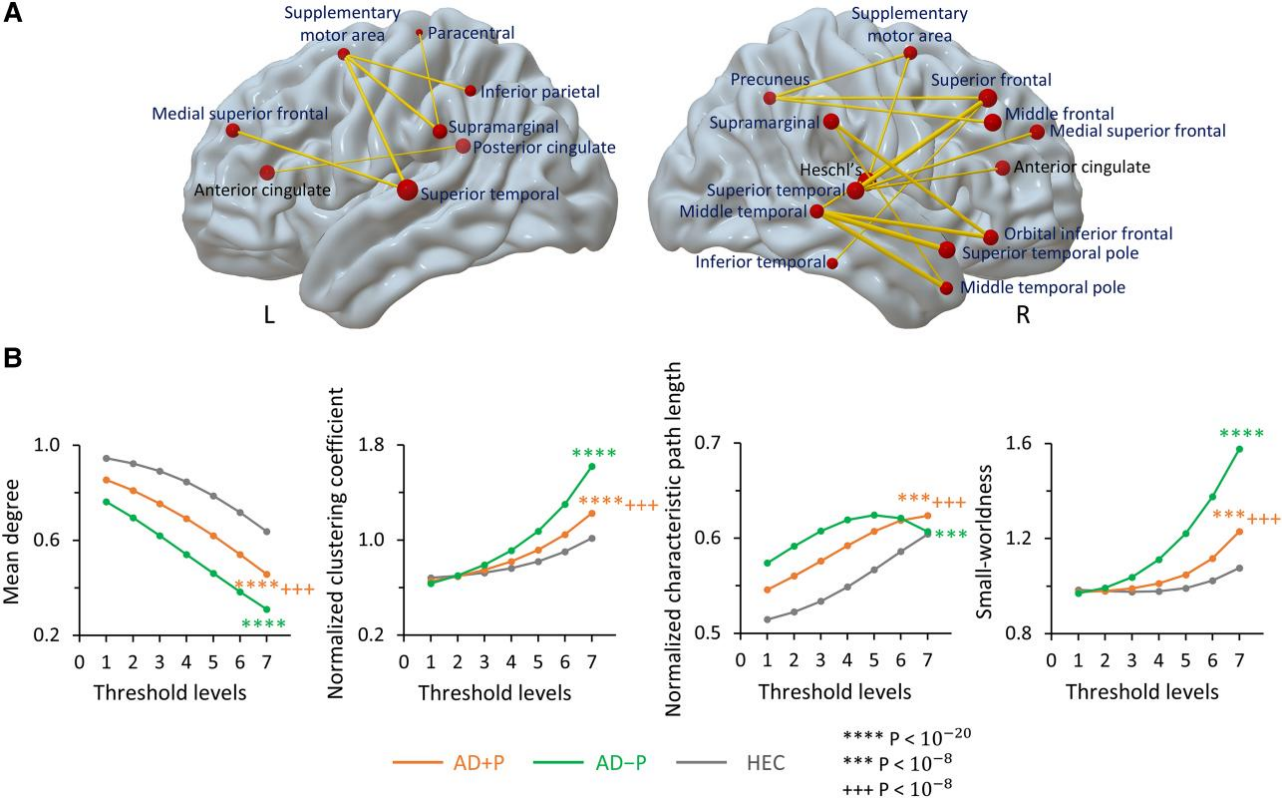
**Alternations in the ADPN network organization**. (**A**) Enhanced connections in AD + P (*N* = 87) relative to AD−P (*N* = 174). Changes in metabolic connectivity between the two groups were validated using bootstrapped data (*N* = 100 per group) and a Student’s *t*-test [T(198) > 21.7, *P* < 2.3×10^−54^), followed by *post hoc* Bonferroni corrections. (**B**) Network metrics including mean degree centrality, clustering coefficient, characteristic path length and small-worldness. These metrics were computed at thresholds ranged from *r* = 0.3 to 0.6, at 0.05 increments. A general linear model for bootstrapped data (*N* = 100 per group) across graph thresholds, followed by *post hoc* Bonferroni tests, was used to evaluate group differences in each network metric. Average network metric of 100 bootstraps were displayed for each group. ****P < 10^−20^, ***P < 10^−8^ relative to HEC, +++ *P* < 10^−8^ relative to AD−P. AD + P, AD with psychosis; AD−P, AD without psychosis; ADPN, Alzheimer’s disease psychosis network; HEC, healthy elderly controls.

Within the ADPN network space, the AD + P group demonstrated enhanced—rather than degraded—metabolic connectivity in comparison with the AD−P group (Fig. 6A, Supplementary Table 7). In this comparison, AD + P evidenced enhanced connections linking (i) the ACC to the auditory cortex (superior temporal gyrus) and posterior cingulate cortex (PCC), (ii) the prefrontal cortex to the precuneus, supramarginal gyrus and temporal cortex, (iii) the SMA to the inferior parietal, precuneus, supramarginal, Heschl’s and superior temporal gyrus and (iv) the middle temporal gyrus to the temporal pole (Fig. 6A, Supplementary Table 7). This augmented connectivity suggests that the emergence of psychosis may result from amplified network communication in Alzheimer’s disease psychosis rather than loss of network function as seen in primary non-neurodegenerative psychotic illness.

To determine the effects of the connectivity changes on network function in AD + P, we quantified graph metrics within the ADPN space. The results for each of the metrics were shown in Fig. 6B. Overall connectivity in the ADPN, measured by mean degree centrality, decreased in both AD + P and AD−P groups compared with HEC (*P* < 10^−20^, corrected). By contrast, characteristic path length (*P* < 10^−20^) increased in the Alzheimer’s disease groups compared with HEC, reflecting less efficient information transfer through the ADPN network. Clustering and small-worldness also increased in the Alzheimer’s disease groups relative to HEC (*P* < 10^−20^), reflecting a higher segregation and a higher ratio of segregation to integration of information sources in the ADPN network. However, when compared with AD−P, AD + P exhibited an increase in degree centrality and a decline in the other metrics (*P* < 10^−8^). These changes were consistent with the gain of connections in AD + P relative to AD−P and the loss of connections in AD + P and AD−P relative to HEC (Supplementary Fig. 3, Supplementary Tables 6 and 8).

## Discussion

In this report, the application of a novel deep learning methodology to the brain FDG PET scans of those who developed psychosis in Alzheimer’s disease has identified a predictive and quantifiable topographical metabolic covariance network, the ADPN. In those with Alzheimer’s disease who developed psychosis, baseline expression of key network regions was higher than in those without psychosis and longitudinal ADPN expression in psychosis continued to increase over time. This is similar to the expression and progression of previously identified disease-specific metabolic networks including non-psychotic Alzheimer’s disease and Parkinson’s disease, whose network dysfunctions tracks with cognitive and motor symptomatology respectively.^20,23,53^ In previous studies, those who experience psychosis in the context of Alzheimer’s disease have been shown consistently to decline more rapidly and evidence greater functional impairment, which are hallmarks of a more aggressive neurodegenerative condition.^7,14,18,52,54,55^ Consonant with this, in the current report, individuals with higher ADPN expression scores evidenced increased impairment on CDR and lower MMSE scores, reflecting the functional and cognitive correlates of the psychotic syndrome.

While the previously reported metabolic network that predicts the cognitive syndrome of Alzheimer’s disease comprises the precuneus and temporoparietal regions,^23^ the ADPN identified in the current report that predicts psychosis in Alzheimer’s disease overlaps in temporoparietal regions but extends to a broader network that includes prefrontal regions and occipital regions encompassing critical structures for visual processing- the lingual and fusiform gyri. As psychosis in Alzheimer’s disease is typified by a more severe cognitive phenotype together with delusions and/or hallucinations that are primarily visual,^2^ the integration of prefrontal cortex with hippocampal/parahippocampal regions and visual cortex in the ADPN represents a logical extension of previous imaging and neurocognitive findings. Imaging studies consistently highlight the critical role of frontal systems in delusions in Alzheimer’s disease.^18,56,57^ Psychosis in Alzheimer’s disease has also been associated with advanced hippocampal and parahippocampal atrophy,^58,59^ a finding that is consistent with the known accelerated cognitive decline that accompanies the behavioural symptoms.^1^ Hallucinations in Alzheimer’s disease have not been as extensively studied in the absence of delusions as perceptual disturbances are less common and predominantly appear together with elaborated delusional ideation, but published studies have implicated the lingual gyrus in particular and the occipital cortex in general.^60,61^ Within the visual cortex, the lingual gyrus is critical for the processing of visual images and provides inputs to the neighbouring fusiform gyrus,^62^ a structure that plays a key role in facial affective processing.^63^ Impaired facial affective processing has been reported in psychotic Alzheimer’s disease,^64^ and the inability to decode the valence of facial emotion from network dysfunction in the visual cortex may contribute to the elaboration of delusions when malevolent intentions are misattributed.

Key regions within the ADPN network space also include nodes not previously implicated in Alzheimer’s disease psychosis, particularly the insula and the SMA. Several lesion studies have highlighted the participation of the insula in psychosis-relevant facial affective processing.^65^ The SMA is a region that is critical to both the planning and execution of voluntary motor behaviours and to the motor aspect of speech production.^66^ As psychosis in Alzheimer’s disease is known to be associated with a poorer quality of life and increased rates of institutionalization due in part to disruptive behaviours that include motor and verbal agitation,^67^ the involvement of the SMA in the ADPN could provide a neurobiological underpinning for this well-known association. Additionally, the SMA plays a critical role in working memory and is a component of the motor-language SMA syndrome.^68^ Working memory deficits have been found to be a defining feature of the cognitive syndrome of psychotic Alzheimer’s disease that have been linked to frontal hypometabolic patterns.^18,52,69^

Within the ADPN space, we found that connectivity between individual nodes was uniformly increased—rather than decreased—in those with Alzheimer’s disease psychosis relative to those with non-psychotic Alzheimer’s disease, predominantly linking frontal with temporal and parietal regions. The SMA was found to be a particularly active node, exhibiting connectivity with structures critical for language and social cognition including Heschl’s gyrus, the superior temporal gyrus, the supramarginal gyrus and the inferior parietal lobule.^70-72^ Alterations in functional connectivity have been previously reported utilizing resting-state functional MRI in primary (non-neurodegenerative) psychotic illnesses. In those with schizophrenia and bipolar disorder, there is strong evidence of disruption of functional network connectivity that include frontoparietal (dorsolateral/dorsomedial prefrontal and lateral parietal), together with the default mode and ventral attention networks.^73,74^ The degradation of the frontoparietal networks in psychotic illness is thought to be reflected in the transdiagnostic dysexecutive cognitive profile observed in psychotic and severe mental illness.^73^ There is also recent evidence of well-defined patterns of disruption of striatal connectivity that comprise increases and decreases in intra- and extra-striatal functional connections reflecting abnormalities of dopaminergic neurotransmission that predict treatment response and may collectively have a value as a diagnostic imaging biomarker in primary psychotic conditions.^75,76^ In non-psychotic Alzheimer’s disease, functional connectivity has generally been reported to be eroded^77^ especially in posterior networks that include the hippocampus,^78^ while connectivity has also been reported to be augmented, especially in networks that include frontal structures.^79-81^ It has been suggested that the trend towards increased frontal connectivity in Alzheimer’s disease may be compensatory for impaired temporal and posterior network coherence.^77^ In Alzheimer’s disease psychosis, in the only published study of a small cohort of Alzheimer’s disease subjects with delusions, reduced connectivity of a single cluster in the inferior parietal lobe that includes the angular gyrus with the other nodes of the default mode network was reported.^82^ In light of the findings in the current report that suggests that psychotic Alzheimer’s disease may be distinguished by increased metabolic connectivity linking nodes with relevance for motor behaviour with regions critical for language and social cognition, it may be that delusions and the aggressive behaviour that follow them in Alzheimer’s disease emerge from compensatory engagement of degraded network structures that are critical for the narrative integration of experience with those regulating behavioural planning and execution.

Although neuropathologic biomarkers that could be relevant to ADPN expression and the cognitive and functional decline associated with it that include quantifications of amyloid and tau pathology were not studied in the current report, Braak stating of tau pathology has previously been found to correlate with the expression of metabolic network patterns in non-psychotic Alzheimer’s disease.^83^ Additionally, several studies have reported that increases in phosphorylated tau pathology in frontal cortex is associated with psychosis in Alzheimer’s disease.^11,12^ In a study of the association of the topography of fibrillar tau pathology with the development of psychosis in Alzheimer’s disease, increases in the retention of the tau PET tracer [^18^F]-AV1451 in frontal regions; precuneus; parahippocampal and supramarginal gyri; and lingual and fusiform gyri that overlap with key regions within the ADPN were observed in those destined to become psychotic over the course of the study.^14^ It follows that tau pathology may be responsible for metabolic abnormalities that adumbrate the ADPN. Further research is required to determine whether individual ADPN expression predicts Alzheimer’s disease neuropathological burden in Alzheimer’s disease psychosis and whether the regional distribution of amyloid and/or tau pathology implicates key ADPN nodes.

While AI has revolutionized the study of disease-related networks in neuroimaging, numerous limitations continue to hinder progress in this field. The application of AI in neuroimaging faces significant challenges,^35,84,85^ including the limited availability of independently annotated real-world datasets for validation, unclear diagnostic criteria and prognosis measures and high computational demands. Additionally, issues with generalization^84,86^ across imaging modalities, ethical concerns^87^ about data privacy and a lack of model transparency^88^—such as the absence of pre-registration and insufficient method descriptions^35^—further restrict its widespread clinical use and trust. Our findings are subject to several limitations. One such limitation is our inability to control for medication status, including antipsychotic medications, cholinesterase inhibitors and NMDA antagonists. Consequently, the derivation of the ADPN may have occurred under varying medication states, potentially influencing the distribution of metabolic activity. Notably, the use of antipsychotic medications could make cognitive decline in psychotic patients more severe,^89^ which may in turn amplify the observed longitudinal increase in ADPN expression in AD + P subjects. Furthermore, the sensitivity of the Neuropsychiatric Inventory-Q to drug-induced behavioural changes, as highlighted by Lai,^90^ suggests that it may not be the most reliable method for establishing psychosis,^89^ thereby raising the possibility of misdiagnosis in some subjects. Lastly, the absence of an independent validation dataset limits the generalizability of our findings. Future studies demonstrating validation in an independent dataset would enhance confidence in the ADPN and support its utilization in a clinical context.

## Conclusion

The application of CNN within an explainable AI framework to FDG PET scan data together with graph theoretical analysis has identified a metabolic network pattern, the ADPN, predictive of psychosis in Alzheimer’s disease and the related accelerated cognitive and functional decline. As there are currently no FDA-approved treatments for psychotic Alzheimer’s disease and currently available antipsychotic medications are associated with an increased risk of mortality,^1^ the development of novel therapies and the identification of those most likely to respond could be facilitated by validated imaging biomarkers that may include the ADPN.

## Supplementary Material

fcaf159_Supplementary_Data

## Data Availability

The authors confirm that the data supporting the findings of this study are available within the article and its Supplementary material. Raw data that support the findings of this study are available on request from the corresponding author, A.V. and N.N., upon request. MATLAB code for the 3D ResNet101 model can be downloaded at https://github.com/vonnlab/ADPN.
