# Supplementary material for: An artificial intelligence-derived metabolic network predicts psychosis in Alzheimer’s disease
Source: Brain Commun. 2025 Apr 25;7(3):fcaf159. doi: 10.1093/braincomms/fcaf159 (PMC12209852; doi:10.1093/braincomms/fcaf159)
Supplement: fcaf159_Supplementary_Data [file fcaf159_Supplementary_Data.docx]

**Supplementary Materials**

**Supplementar**y **Table 1.** Parameters of the proposed 3D residual neural network

| **Parameter** | **Type/Value** |
| --- | --- |
| Optimizer | Sgdm |
| Execution environment | multi-gpu |
| Initial learning rate | 0.01 |
| L2 Regularization | 0.0005 |
| Gradient threshold | 0.05 |
| Max epochs | 1000 |
| Shuffle | Every epoch |
| Validation frequency | 30 |
| Mini batch size | 32 |
| Drop ​​rate | 0.2 |

**Supplementar**y **Table 2.** Confusion matrix and performance metrics of the ADPN and conventional PET-based classifiers on the prediction of AD+P and HEC from the testing dataset


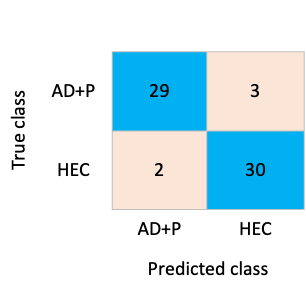

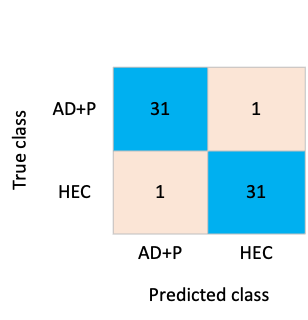


**ADPN classifier**

**Conventional classifier**

|  | **ADPN classifier** | **Conventional classifier** |
| --- | --- | --- |
| Accuracy (%) | 96.9 | 92.2 |
| Sensitivity (%) | 96.9 | 90.6 |
| Specificity (%) | 96.9 | 93.4 |
| Precision (%) | 96.9 | 93.6 |
| F1 score | 0.97 | 0.92 |

**Supplementar**y **Table 3.**

| **Brain regions specific to HEC (Expression score in HEC > AD+P)** | | |
| --- | --- | --- |
| **Brain region** | **Abbreviation** | **AAL ID** |
| Precentral_L | PreC | 1 |
| Rolandic_Oper_L | ROL | 17 |
| Frontal_Sup_Medial_LR | SFmed | 23-24 |
| Cingulum_Ant_LR | AC | 31-32 |
| Cingulum_Mid_L | MC | 33 |
| Cingulum_Post_L | PC | 35 |
| Occipital_Sup_L | SO | 49 |
| Occipital_Mid_L | MO | 51 |
| Postcentral_L | PoC | 57 |
| Parietal_Sup_LR | SP | 59-60 |
| Parietal_Inf_L | IP | 61 |
| SupraMarginal_L | SMG | 63 |
| Angular_L | ANG | 65 |
| Precuneus_LR | PCUN | 67-68 |
| Paracentral_Lobule_LR | PCL | 69-70 |
| Heschl_L | HES | 79 |
| Temporal_Sup_L | ST | 81 |
| Temporal_Mid_L | MT | 85 |

HEC, normal control; AD+P, AD with psychosis; AAL, automated anatomical labeling brain atlas^42^; L, left; R, right.

**Supplementar**y **Table 4.** Confusion matrix and performance metrics of the ADPN- and conventional PET-based classifiers on the prediction of AD+P and AD−P using 5-fold cross-validation.


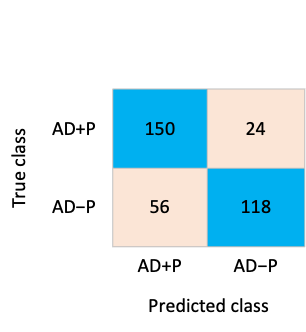

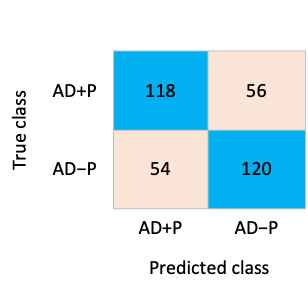


**ADPN-based classifier**

**Conventional classifier**

|  | **ADPN-based classifier** | **Conventional classifier** |
| --- | --- | --- |
| Accuracy (%) | 77.0 | 68.4 |
| Sensitivity (%) | 86.2 | 67.8 |
| Specificity (%) | 67.8 | 69.0 |
| Precision (%) | 72.8 | 68.6 |
| F1 score | 0.79 | 0.68 |

**Supplementar**y **Table 5.** Brain regions specific to the difference between AD+P (n = 87) and AD−P (n = 174) within the ADPN that exhibit a significant difference in their expression scores at baseline.

| **AAL ID** | **Brain region** | **AD+P** | | | **AD−P** | | | **^a^P-value** |
| --- | --- | --- | --- | --- | --- | --- | --- | --- |
| 01 | Precentral_L | ^b^8.54 | ± | 2.88 | 6.46 | ± | 3.95 | 0.002 |
| 02 | Precentral_R | 11.17 | ± | 2.48 | 9.40 | ± | 3.86 | 0.013 |
| 03 | Frontal_Sup_L | 10.20 | ± | 3.13 | 7.94 | ± | 4.23 | 0.002 |
| 04 | Frontal_Sup_R | 11.73 | ± | 3.03 | 9.47 | ± | 4.34 | 0.002 |
| 07 | Frontal_Mid_L | 7.36 | ± | 2.24 | 5.67 | ± | 3.09 | 0.001 |
| 08 | Frontal_Mid_R | 8.95 | ± | 1.83 | 7.46 | ± | 2.89 | 0.002 |
| 11 | Frontal_Inf_Oper_L | 3.30 | ± | 1.19 | 2.40 | ± | 1.65 | 0.001 |
| 13 | Frontal_Inf_Tri_L | 2.85 | ± | 1.02 | 2.13 | ± | 1.32 | 0.001 |
| 17 | Rolandic_Oper_L | 2.59 | ± | 1.08 | 1.82 | ± | 1.52 | 0.003 |
| 19 | Supp_Motor_Area_L | 18.66 | ± | 5.90 | 14.67 | ± | 7.82 | 0.004 |
| 20 | Supp_Motor_Area_R | 18.28 | ± | 5.58 | 14.51 | ± | 7.51 | 0.005 |
| 23 | Frontal_Sup_Medial_L | 6.94 | ± | 2.00 | 5.41 | ± | 2.80 | 0.001 |
| 24 | Frontal_Sup_Medial_R | 7.15 | ± | 1.93 | 5.63 | ± | 2.76 | 0.001 |
| 29 | Insula_L | 3.35 | ± | 1.42 | 2.56 | ± | 1.77 | 0.039 |
| 31 | Cingulum_Ant_L | 4.92 | ± | 1.52 | 3.87 | ± | 2.23 | 0.009 |
| 32 | Cingulum_Ant_R | 5.08 | ± | 1.51 | 4.04 | ± | 2.23 | 0.010 |
| 33 | Cingulum_Mid_L | 8.94 | ± | 2.80 | 6.90 | ± | 4.33 | 0.008 |
| 34 | Cingulum_Mid_R | 9.68 | ± | 2.72 | 7.64 | ± | 4.34 | 0.008 |
| 57 | Postcentral_L | 4.76 | ± | 1.80 | 3.43 | ± | 2.57 | 0.002 |
| 61 | Parietal_Inf_L | 2.17 | ± | 1.07 | 1.41 | ± | 1.64 | 0.011 |
| 63 | SupraMarginal_L | 2.03 | ± | 0.93 | 1.27 | ± | 1.40 | 0.001 |
| 65 | Angular_L | 1.24 | ± | 0.73 | 0.74 | ± | 1.16 | 0.029 |
| 69 | Paracentral_Lobule_L | 11.44 | ± | 4.18 | 8.78 | ± | 5.56 | 0.010 |
| 70 | Paracentral_Lobule_R | 9.46 | ± | 3.48 | 7.29 | ± | 4.79 | 0.021 |
| 79 | Heschl_L | 2.91 | ± | 1.25 | 2.10 | ± | 1.72 | 0.014 |
| 81 | Temporal_Sup_L | 2.35 | ± | 1.09 | 1.65 | ± | 1.35 | 0.004 |
| 85 | Temporal_Mid_L | 2.72 | ± | 1.29 | 2.04 | ± | 1.35 | 0.011 |

AD+P, AD with psychosis; AD−P, AD without psychosis; AAL, automated anatomical labeling brain atlas^42^; L, left; R, right. ^a^2-tailed Student’s *t* test with Bonferroni correction for multiple comparisons. ^b^All the data are presented as mean $\pm$ SD of expression scores in each brain region.

**Supplementar**y **Table 6.** Metabolic connectivity reduced in AD+P relative to HEC subjects.

| **n** | **ROI 1** | **ROI 2** | **r HEC** | **r AD+P** | **P-value** |
| --- | --- | --- | --- | --- | --- |
| 1 | Precentral_L | Occipital_Sup_L | 0.77 | 0.51 | 3.4E-82 |
| 2 | Precentral_L | Occipital_Mid_L | 0.75 | 0.50 | 3.9E-80 |
| 3 | Rolandic_Oper_L | Occipital_Sup_L | 0.76 | 0.50 | 1.6E-76 |
| 4 | Rolandic_Oper_L | Occipital_Mid_L | 0.79 | 0.51 | 1.3E-86 |
| 5 | Frontal_Sup_Medial_L | Parietal_Sup_L | 0.66 | 0.45 | 1.3E-72 |
| 6 | Cingulum_Ant_L | Fusiform_L | 0.68 | 0.47 | 4.6E-79 |
| 7 | Cingulum_Ant_L | Angular_L | 0.67 | 0.45 | 7.1E-76 |
| 8 | Cingulum_Ant_L | Temporal_Mid_L | 0.72 | 0.52 | 1.0E-78 |
| 9 | Cingulum_Post_L | Vermis | 0.69 | 0.45 | 7.8E-75 |
| 10 | Hippocampus_L | Occipital_Sup_L | 0.61 | 0.35 | 5.1E-67 |
| 11 | Hippocampus_L | Occipital_Mid_L | 0.70 | 0.41 | 3.7E-86 |
| 12 | Hippocampus_L | Cerebelum_R | 0.60 | 0.40 | 1.9E-53 |
| 13 | ParaHippocampal_L | Occipital_Mid_L | 0.64 | 0.32 | 4.7E-85 |
| 14 | ParaHippocampal_L | Angular_L | 0.63 | 0.42 | 3.3E-63 |
| 15 | ParaHippocampal_L | Cerebelum_R | 0.60 | 0.39 | 4.8E-51 |
| 16 | Amygdala_L | Occipital_Mid_L | 0.62 | 0.25 | 4.7E-93 |
| 17 | Amygdala_L | Angular_L | 0.64 | 0.40 | 8.8E-73 |
| 18 | Occipital_Sup_L | Postcentral_L | 0.81 | 0.53 | 2.6E-94 |
| 19 | Occipital_Mid_L | Postcentral_L | 0.78 | 0.50 | 2.5E-89 |
| 20 | Fusiform_R | Vermis | 0.61 | 0.36 | 1.8E-64 |
| 21 | Precentral_R | Lingual_R | 0.58 | 0.27 | 4.1E-67 |
| 22 | Frontal_Mid_R | Lingual_R | 0.55 | 0.10 | 6.6E-97 |
| 23 | Frontal_Inf_Oper_R | Lingual_R | 0.57 | 0.13 | 4.9E-95 |
| 24 | Rolandic_Oper_R | Lingual_R | 0.58 | 0.28 | 3.1E-75 |
| 25 | Lingual_R | Heschl_R | 0.56 | 0.32 | 3.5E-62 |
| 26 | Lingual_R | Temporal_Sup_R | 0.58 | 0.36 | 8.0E-60 |

**Supplementar**y **Table 7.** Metabolic connectivity enhanced in AD+P relative to AD−P.

| **n** | **ROI 1** | **ROI 2** | **r AD−P** | **r AD+P** | **P-value** |
| --- | --- | --- | --- | --- | --- |
| 1 | Frontal_Sup_R | Precuneus_R | 0.40 | 0.66 | 4.3E-75 |
| 2 | Frontal_Sup_R | Temporal_Sup_R | 0.52 | 0.74 | 2.5E-71 |
| 3 | Frontal_Sup_R | Temporal_Mid_R | 0.39 | 0.64 | 1.9E-69 |
| 4 | Frontal_Sup_R | Temporal_Inf_R | 0.41 | 0.61 | 2.3E-54 |
| 5 | Frontal_Mid_R | Precuneus_R | 0.43 | 0.65 | 5.8E-60 |
| 6 | Frontal_Inf_Orb_R | SupraMarginal_R | 0.48 | 0.68 | 4.5E-65 |
| 7 | Frontal_Inf_Orb_R | Temporal_Mid_R | 0.44 | 0.71 | 7.1E-74 |
| 8 | Supp_Motor_Area_L | Parietal_Inf_L | 0.43 | 0.64 | 1.4E-82 |
| 9 | Supp_Motor_Area_L | SupraMarginal_L | 0.42 | 0.66 | 1.5E-91 |
| 10 | Supp_Motor_Area_L | Temporal_Sup_L | 0.42 | 0.67 | 8.1E-90 |
| 11 | Supp_Motor_Area_R | Precuneus_R | 0.46 | 0.66 | 1.9E-69 |
| 12 | Supp_Motor_Area_R | Heschl_R | 0.42 | 0.64 | 8.5E-70 |
| 13 | Frontal_Sup_Medial_L | Temporal_Sup_L | 0.43 | 0.65 | 6.7E-71 |
| 14 | Frontal_Sup_Medial_R | Temporal_Sup_R | 0.42 | 0.66 | 8.3E-61 |
| 15 | Cingulum_Ant_L | Cingulum_Post_L | 0.40 | 0.61 | 2.8E-63 |
| 16 | Cingulum_Ant_R | Temporal_Sup_R | 0.41 | 0.64 | 2.2E-56 |
| 17 | SupraMarginal_L | Paracentral_Lobule_L | 0.40 | 0.60 | 2.5E-78 |
| 18 | SupraMarginal_R | Temporal_Pole_Mid_R | 0.39 | 0.61 | 1.0E-71 |
| 19 | Temporal_Pole_Sup_R | Temporal_Mid_R | 0.53 | 0.75 | 1.7E-83 |
| 20 | Temporal_Mid_R | Temporal_Pole_Mid_R | 0.46 | 0.73 | 1.0E-88 |

**Supplementar**y **Table 8.** Metabolic connectivity reduced for AD−P relative to HEC subjects.

| **n** | **ROI 1** | **ROI 2** | **r HEC** | **r AD-P** | **P-value** |
| --- | --- | --- | --- | --- | --- |
| 1 | Frontal_Sup_R | Precuneus_R | 0.79 | 0.40 | 3.7E-115 |
| 2 | Frontal_Inf_Tri_R | Precuneus_R | 0.72 | 0.36 | 8.9E-102 |
| 3 | Frontal_Inf_Orb_R | Precuneus_R | 0.61 | 0.23 | 9.1E-101 |
| 4 | Frontal_Inf_Orb_R | Temporal_Mid_R | 0.85 | 0.44 | 1.0E-123 |
| 5 | Frontal_Sup_Medial_L | Parietal_Sup_L | 0.66 | 0.18 | 5.5E-123 |
| 6 | Frontal_Sup_Medial_L | Parietal_Inf_L | 0.73 | 0.28 | 6.4E-126 |
| 7 | Frontal_Sup_Medial_L | SupraMarginal_L | 0.75 | 0.34 | 3.2E-123 |
| 8 | Frontal_Sup_Medial_L | Angular_L | 0.72 | 0.23 | 1.2E-131 |
| 9 | Frontal_Sup_Medial_L | Precuneus_L | 0.67 | 0.23 | 1.3E-118 |
| 10 | Frontal_Sup_Medial_L | Temporal_Mid_L | 0.72 | 0.30 | 8.3E-121 |
| 11 | Frontal_Sup_Medial_R | Parietal_Sup_R | 0.60 | 0.19 | 2.0E-107 |
| 12 | Frontal_Sup_Medial_R | SupraMarginal_R | 0.72 | 0.37 | 4.2E-110 |
| 13 | Frontal_Sup_Medial_R | Precuneus_R | 0.68 | 0.22 | 1.6E-122 |
| 14 | Frontal_Sup_Medial_R | Temporal_Mid_R | 0.71 | 0.25 | 9.1E-111 |
| 15 | Cingulum_Ant_L | Fusiform_L | 0.68 | 0.31 | 3.1E-97 |
| 16 | Cingulum_Ant_L | Parietal_Inf_L | 0.67 | 0.22 | 1.2E-111 |
| 17 | Cingulum_Ant_L | SupraMarginal_L | 0.72 | 0.31 | 1.7E-113 |
| 18 | Cingulum_Ant_L | Angular_L | 0.67 | 0.17 | 1.3E-121 |
| 19 | Cingulum_Ant_L | Temporal_Mid_L | 0.72 | 0.29 | 1.8E-119 |
| 20 | Cingulum_Ant_R | Fusiform_R | 0.65 | 0.28 | 2.6E-86 |
| 21 | Cingulum_Ant_R | SupraMarginal_R | 0.69 | 0.31 | 8.2E-108 |
| 22 | Cingulum_Ant_R | Precuneus_R | 0.60 | 0.13 | 3.1E-108 |
| 23 | Cingulum_Ant_R | Temporal_Mid_R | 0.69 | 0.22 | 1.7E-113 |
| 24 | Cingulum_Ant_R | Temporal_Inf_R | 0.65 | 0.29 | 3.8E-90 |
| 25 | Amygdala_L | Occipital_Mid_L | 0.62 | 0.22 | 2.6E-101 |
| 26 | Amygdala_L | Angular_L | 0.64 | 0.28 | 1.3E-97 |

**
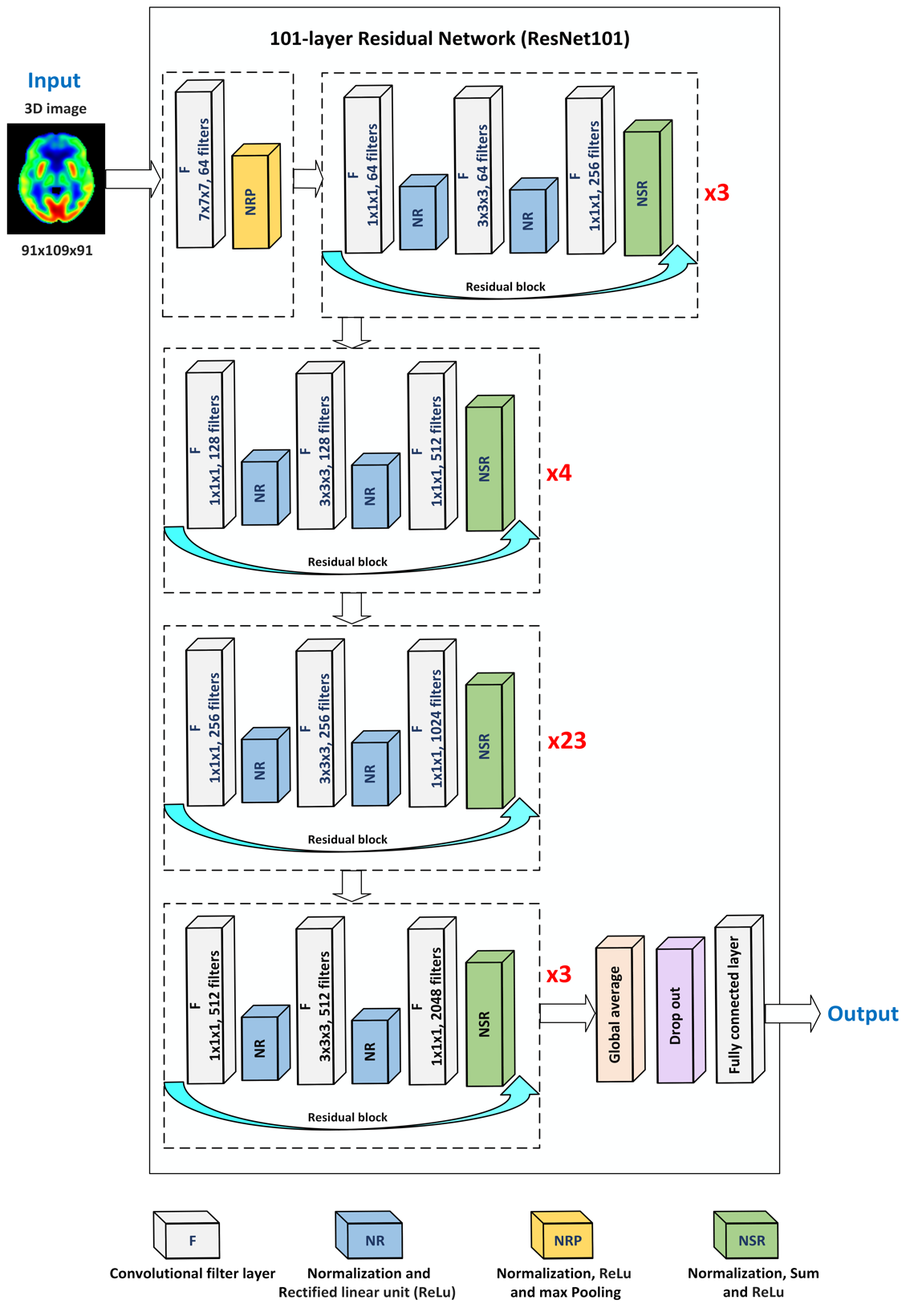
Supplementar**y **Figure 1.** The 3D ResNet101 network architecture. The network starts with a **convolutional layer** followed by **max pooling**, then consists of **residual blocks** arranged in four stages, with increasing numbers of filters (64, 128, 256, and 512). After the residual blocks, it uses **global average pooling** to reduce the feature map size, dropout to reduce the overfitting of neural networks and a **fully connected layer** to output the final classification result.


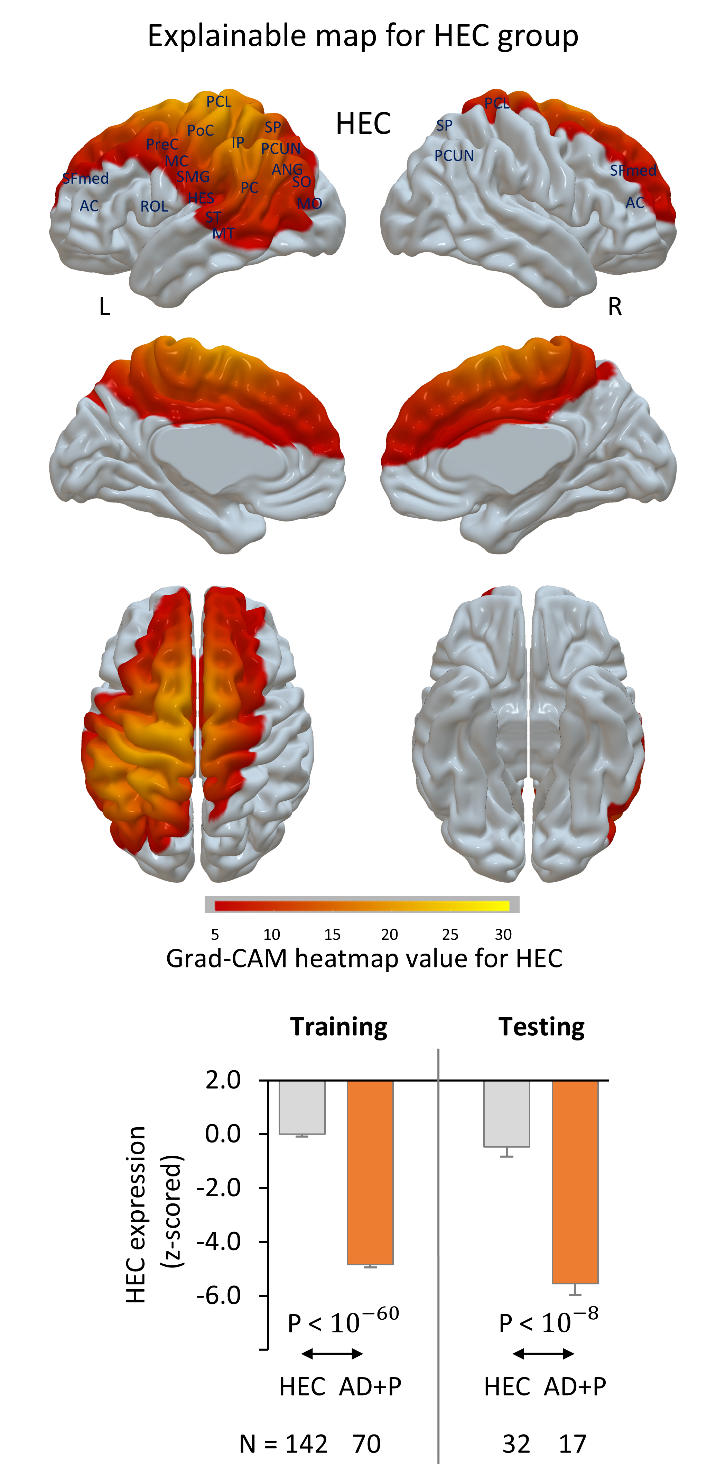
**Supplementar**y **Figure 2.** Explainable map for HEC group. The HEC expression score, the average of expression scores across regions specific to HEC group, exhibited an elevation in HEC compared to the AD+P patients (training set: T(210) = 34.9, P <${10}^{-60}$; testing set: T(47) = 8.7, p <${10}^{-8}$). Heatmap values are unitless and scaled for visualization. Student’s t-test was used to examine the difference in expression scores between the two groups. AC, anterior cingulum; ANG, angular; HES, Heschl; IP, inferior parietal; MC, middle cingulum; MO, middle occipital; MT, middle temporal; PC, posterior cingulum; PCL, paracentral lobule; PCUN, precuneus; PoC, postcentral; PreC, precentral; ROL, rolandic operculum; SFmed, medial superior frontal; SMG, supramarginal; SO, superior occipital; SP, superior parietal; ST, superior temporal; L, left; R, right; Grad-CAM, gradient-weighted class activation.


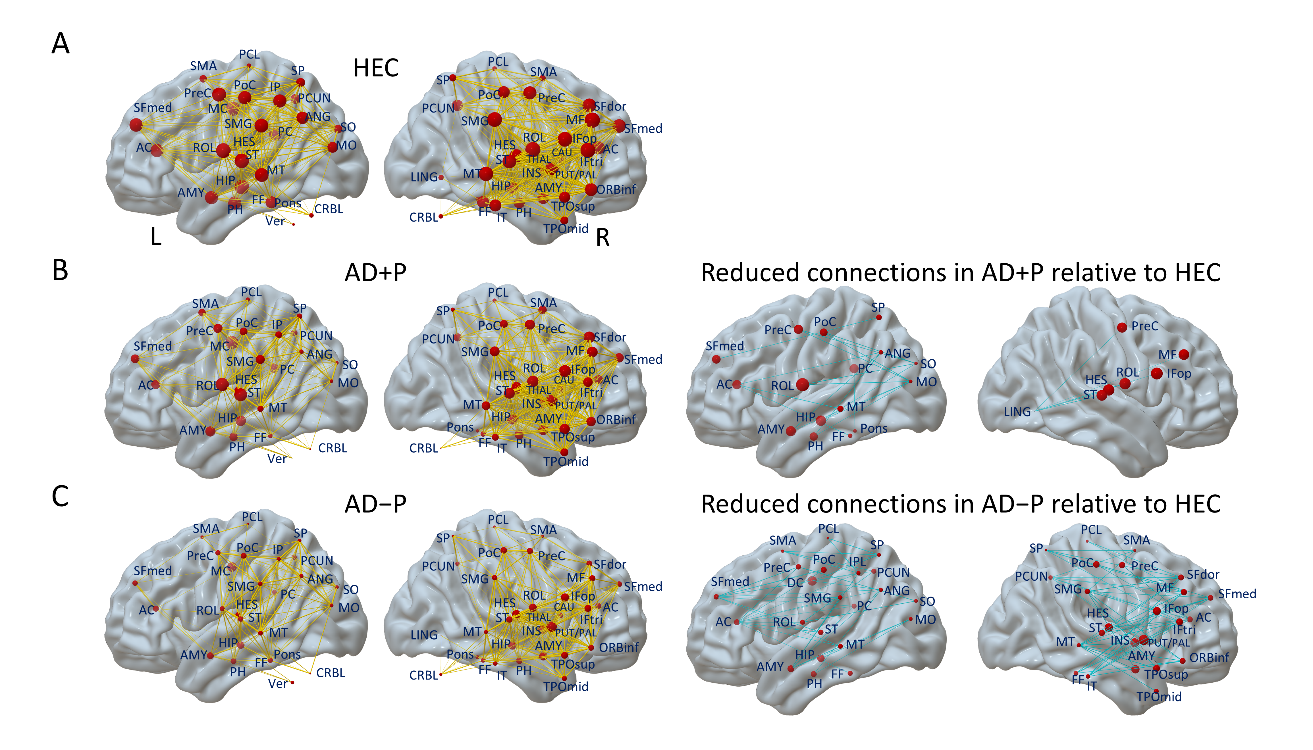
**Supplementar**y **Figure 3.** Reduced metabolic connectivity in AD+P (N = 87) and AD−P (N = 174) relative to HEC (N = 174) within the ADPN network. ADPN regions are represented by spheres with radius proportional to the corresponding nodal degree from each group. Connections linking pairs of nodes are represented by yellow lines with thickness proportional to the corresponding correlation coefficient (*r*) from each group. Reduced connections linking pairs of nodes are represented by cyan lines with thickness proportional to the corresponding correlation coefficient (*r*) from each AD group. Changes in metabolic connectivity between the two groups were validated using bootstrapped data (N = 100 per group) and a Student’s t-test (T(198) > 20.6, P < 4.8E-51), followed by post-hoc Bonferroni corrections.

| **Abbreviations** | **AAL brain regions** | **Abbreviations** | **AAL brain regions** |
| --- | --- | --- | --- |
| AC | Cingulum_Ant_LR | PC | Cingulum_Post_L |
| AMY | Amygdala_LR | PCL | Paracentral_Lobule_LR |
| ANG | Angular_L | PCUN | Precuneus_LR |
| CAU | Caudate_R | PH | ParaHippocampal_LR |
| CRBL | Cerebellum_LR | PoC | Postcentral_LR |
| FF | Fusiform_LR | Pons | Pons_LR |
| HES | Heschl_LR | PreC | Precentral_LR |
| HIP | Hippocampus_LR | PUT | Putamen_R |
| IFop | Frontal_Inf_Oper_R | ROL | Rolandic_Oper_LR |
| IFtri | Frontal_Inf_Tri_R | SF | Frontal_Sup_R |
| INS | Insula_R | SFmed | Frontal_Sup_Medial_LR |
| IP | Parietal_Inf_L | SMA | Supp_Motor_Area_LR |
| IT | Temporal_Inf_R | SMG | SupraMarginal_LR |
| LING | Lingual_R | SO | Occipital_Sup_L |
| MC | Cingulum_Mid_L | SP | Parietal_Sup_LR |
| MF | Frontal_Mid_R | ST | Temporal_Sup_LR |
| MO | Occipital_Mid_L | THAL | Thalamus_R |
| MT | Temporal_Mid_LR | TPOmid | Temporal_Pole_Mid_R |
| ORBinf | Frontal_Inf_Orb_R | TPOsup | Temporal_Pole_Sup_R |
| PAL | Pallidum_R | Ver | Vermis |
